# Mocha *tyrosinase* variant: a new flavour of cat coat coloration

**DOI:** 10.1111/age.12765

**Published:** 2019-02-04

**Authors:** Y. Yu, R. A. Grahn, L. A. Lyons

**Affiliations:** ^1^ Department of Clinical Veterinary Medicine Nippon Veterinary and Life Science University Musashino Tokyo 180‐8602 Japan; ^2^ Department of Veterinary Medicine and Surgery College of Veterinary Medicine University of Missouri – Columbia Columbia MO 65201 USA; ^3^ Veterinary Genetics Laboratory School of Veterinary Medicine University of California – Davis Davis CA 95616 USA

**Keywords:** Burmese, feline, felis, Thailand, *TYR*

## Abstract

A novel coloration named ‘mocha’ has been identified in the Burmese cat breed from Thailand. *Tyrosinase* (*TYR*) mutations are known to be associated with coat coloration in cats, such as the sable Burmese, the points of the Siamese and albino cats. Additionally, sable Burmese that produced mocha‐colored cats had unexpected genotypes for *TYR*. Therefore, *TYR* was considered a candidate gene for mocha in cats. Sanger sequencing for genomic DNA revealed NC_018732.3:chromosome D1:45 898 609_45 898 771dup in exon 2 and intron 2 of *TYR*. Transcription analysis using cDNA detected c.820_936delinsAATCTC (p.I274_L312delinsNL), which caused a 111‐bp (37 amino acid) deletion in the reading frame of *TYR*. The identified variant was concordant with the phenotype and segregated with *TYR* variants in a pedigree of 12 Burmese cats. This findings of this study suggest that *TYR* is associated with the mocha coloration in cats. The new color variant adds to the allelic series for *TYR* (*C* >* c* ^*b*^ =* c* ^*s*^ >* c*,* c* ^2^) and is recessive to full color (*C*); however, interactions with the *c* ^*b*^ and *c* ^*s*^ alleles are unclear due to the temperature‐sensitivity of the alleles.

The *color* locus (*C*) in mammals is the gene *tyrosinase* (*TYR*), which codes for an enzyme in melanin synthesis. Cats have *TYR* variants causing the temperature‐sensitive colorations of the sable Burmese (*c* ^*b*^ *c* ^*b*^) and the points of the Siamese (*c* ^*s*^ *c* ^*s*^) (Lyons *et al*. 2005; Schmidt‐Küntzel *et al*. 2005; OMIA 000202‐9685). Both breeds originated in Thailand and are known as the Suphalak (Burmese) and Wichien‐maat (Siamese) (Clutterbuck 2004). Two complete albino variants (*c*,* c* ^2^) are also known in *TYR* for cats (Imes *et al*. 2006; Abitbol *et al*. 2017; OMIA 000202‐9685). A vast number of albinism variants are known for *TYR* in various species, including loss‐of‐function variants that cause oculocutaneous albinism Type 1A (OCA1A; OMIM: 203100) and variants that reduce enzymatic activity, causing oculocutaneous albinism Type 1B (OCA1B; OMIM: 606952) in humans. Similar temperature‐sensitive phenotypes have been noted in the Himalayan mouse (Kwon *et al*. 1989), rabbit (Aigner *et al*. 2000), gerbil (Petrij *et al*. 2001), mink (Benkel *et al*. 2009) and human (Giebel *et al*. 1991).

Recent breedings of Burmese cats have revealed an unusual color, termed ‘mocha’ (Fig. 1). Commercial genetic testing of mocha‐colored cats revealed wildtype alleles (*CC*) for all the *TYR* variants. Sable‐colored Burmese cats that have produced mocha kittens, which should be homozygous *c* ^*b*^ *c* ^*b*^, have genotyped as *Cc* ^*b*^. Thus, a novel allele in *TYR*, with the allelic designation *c* ^*m*^, has been suspected to produce the new coloration. In the current study, *TYR* was investigated as a candidate gene for the novel mocha phenotype identified in Burmese cats that have recent Thai origins.

**Figure 1:**
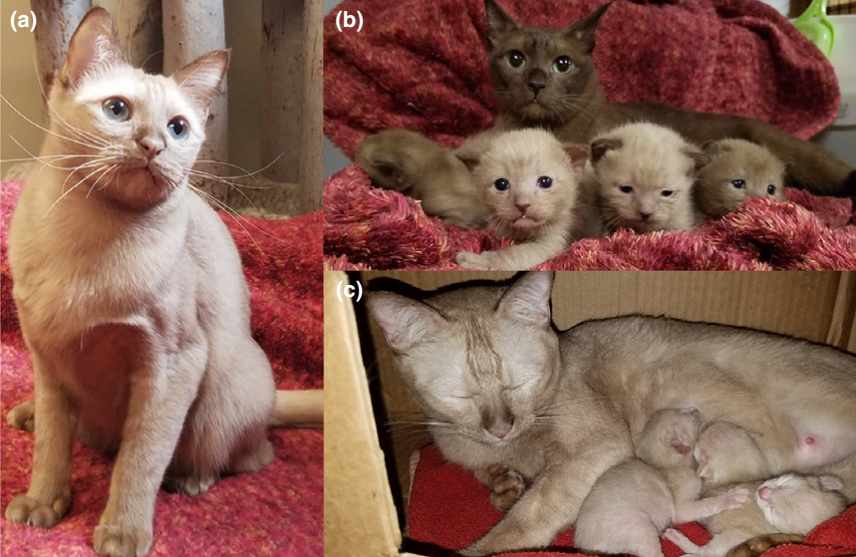
Mocha coloration in Burmese cats (a) Mocha coloration of an intact adult female (proband). This cat has the genotype *aa*,*BB*,*CC*,*DD* by commercial genetic testing, suggesting its coloration should be solid black. Mocha cats have aqua eye coloration and pink nose leather and paw pads. (b) A sable queen (*c* ^*b*^ *c* ^*m*^) with two sable kittens (*c* ^*b*^ *c* ^*b*^; left and right) and two mocha carrier kittens (middle). The two lighter kittens were identified as heterozygous carriers of the Burmese and the newly discovered *TYR* ‘mocha’ variant (*c* ^*b*^ *c* ^*m*^), suggesting that the allele is temperature‐sensitive and co‐dominant with the Burmese (*c* ^*b*^) allele. (c) The same mocha cat as in (a) and her kittens, whose sire is a seal point Siamese (*aa, B‐, c* ^*s*^ *c* ^*s*^). These cats are suspected to have both the mocha and Siamese alleles (*c* ^*m*^ *c* ^*s*^). Siamese kittens (*c* ^*s*^ *c* ^*s*^) would be nearly white with only black ears, paws, face and paws (points). Although the DNA of these cats have not been directly examined, based on parentage, they are inferred to be compound heterozygotes for both the mocha and Siamese alleles (*c* ^*m*^ *c* ^*s*^).

Detailed materials and methods are provided in Appendix S1; a list of primers is provided in Table S1. Five offspring and the proband mocha cat were ascertained for direct Sanger sequencing of the coding regions and the splice junctions of *TYR* (Figs. 1 & S1). A pedigree representing the breeding that produced a mocha colored cat is presented in Fig. S1. The parentage of the extended pedigree was confirmed using short tandem repeat polymorphisms (data not shown). All five *TYR* exons and the intron exon boundaries were amplified in 11 cats, including six cats in the pedigree (Fig. S1) and five unrelated non‐mocha Burmese cats from the US. Amplification of exon 2 (688 bp) produced a second amplicon of approximately 851 bp in five of 11 tested Burmese cats. One cat was homozygous for the larger amplicon (Fig. S2a), which was the mocha proband (Figs. 1a & S1). The approximate 851‐bp product was confirmed by Sanger sequencing as a tandem duplication containing 100 bp of the 3′ portion of exon 2 and 63 bp of the 5′ portion of intron 2 (NC_018732.3:45 898 609–45 898 771dup; Fig. 2). Sequence analyses also identified known single nucleotide variants within the *TYR* exons (Table S2). The known Burmese allelic variant, c.679G>T (p.Gly227Trp) in exon 1, was compound heterozygous with the mocha variant in tested cats (Fig. S1, Table S2). The mocha cat with the homozygous variant did not have the Burmese allele c.679G>T (p.Gly227Trp) nor any other known *TYR* variants causing alterations in coloration.

**Figure 2:**
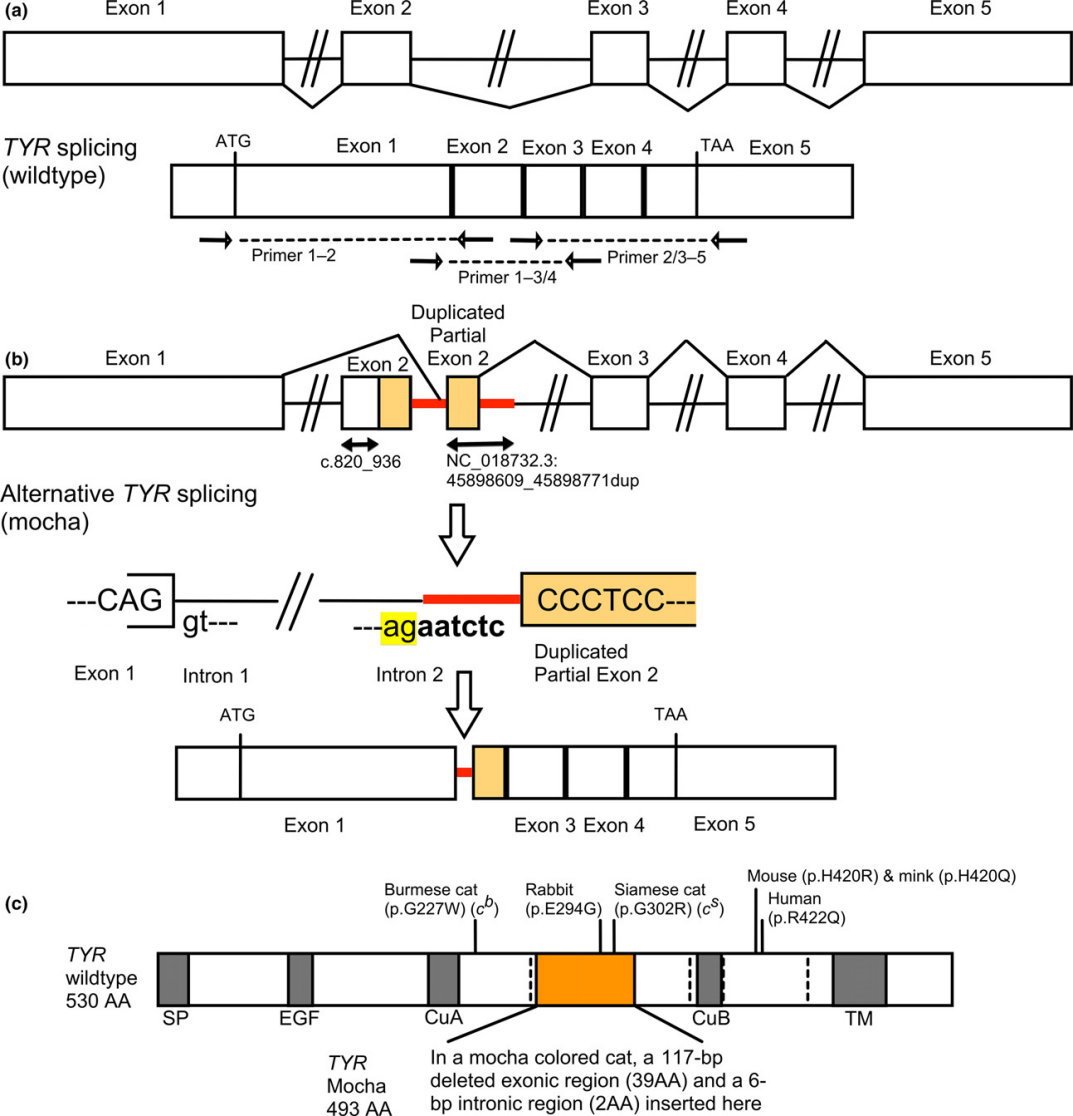
*Tyrosinase (TYR*) exon 2 tandem duplication in cats. (a) Schematic illustration of the *TYR* structure in genomic DNA and complementary DNA (cDNA) of a wildtype cat. Primer positions for RT‐PCR are indicated. (b) Splicing alteration in the mocha coated cat's cDNA is indicated. Duplicated areas of partial exon 2 are indicated by mocha‐colored blocks. Duplicated intronic regions are shown by solid red lines. Duplicated positions of partial exon 2 and the intronic region are indicated as NC_018732.3:45 898 609_45 898 771dup and by the bidirectional arrow. In the splicing process of *TYR* of the mocha‐coated cat, the normal exon 2 was skipped; however, the duplicated partial exon 2 and intronic 6 bp upstream (shown as red bold) are demonstrated to be transcribed due to the presence of the novel splice acceptor site (ag) (highlighted in yellow). The cDNA deletion region is shown by the bidirectional arrow and indicated as c.820_936. (c) Schematic illustration of cDNA *TYR* structure in deduced amino acids of a wildtype cat and a mocha‐coated cat. The domains are colored gray and annotated as SP (signal peptide), EGF (epidermal growth factor‐like domain), CuA (copper‐binding domain A), CuB (copper‐binding domain B) and TM (transmembrane domain). Several temperature‐sensitive *TYR* variants in mammals, including cats, are also annotated, and bars below each allelic designation is indicated by a black bar. The deleted region found in a mocha cat is colored orange. The dotted lines represent boundaries of each exon.

Six cats of the pedigree, which included four half‐siblings of the proband and their parents (Fig. S1), were genotyped for exon 2 by fragment analysis on an agarose gel. The parents and two of the kittens were heterozygous for mocha and the other two kittens were wildtype (Fig. 1b). The mocha variant was genotyped in 20 Khao Manee cats, five Thai cats and 28 random‐bred cats from Thailand (Table S3). All cats were wildtype except two random‐bred cats genotyped as heterozygous for the mocha variant, representing an allele frequency of 1.89% in cats from Thailand.

Complementary DNA (cDNA) analysis from a skin biopsy of the mocha proband was examined to determine the effect of the identified variant on the *TYR* transcript. The reverse transcription PCR (RT‐PCR) amplicon that included the duplicated region of a mocha cat was 875 bp, whereas the amplicon of the control cat was a 986 bp (Fig. S2b). Sequence analysis of the cDNA suggested that the mocha cat's *TYR* splicing pattern is altered, as presented in Fig. 2. In a mocha cat, the wildtype exon 2 is not transcribed. The exon 1 donor site (gt) is spliced to the novel splice acceptor site (ag), which is 7–8 bp upstream of the duplicated portion of the exon 2 sequence (Fig. 2), suggesting a greater splicing affinity than the normal exon 2 acceptor site (ag) and leading to exon 2 skipping. Six nucleotides of the intronic region are included within the altered transcript with the duplicated portion of exon 2, retaining the normal reading frame. The normal and intact splice‐donor site at the 3′ end of the duplicated portion of exon 2 is spliced to the splice‐acceptor of exon 3, staying in frame. Therefore, the variant is a 117‐bp deletion concurrent with a 6‐bp alternative transcription resulting in the altered transcript c.820_936delinsAATCTC (p.Ile274_Leu312delinsAsnLeu) (DDBJ accession no. LC424926). Comparison of known *TYR* variants in cats and other mammals and the nucleotide alignment are depicted in Figs. 2 & S3. [Please note, the sequence annotation of the latest feline genome assembly (Felis_catus_9.0; *Felis catus* Annotation Release 104) is incorrect for *TYR* and is clarified in Appendix S2.]

Burmese cats have low genetic diversity and high inbreeding, which has led to a prevalence of several diseases in the population, such as periodic hypokalemic polymyopathy, frontonasal dysplasia, diabetes and feline orofacial pain syndrome (Rand *et al*. 1997; Rusbridge *et al*. 2010; Gandolfi *et al*. 2012; Lyons *et al*. 2016). The novel mocha coat coloration was identified as a consequence of importation of street cats from Thailand into the Burmese breed to improve genetic diversity. ‘Mocha’ refers to a chocolate‐flavored variant of a caffè latte (Campbell & Smith 1993), and the term etymologically derives from Arabic, referring to Mocha, Yemen, a port on the Red Sea, which was a major marketplace for coffee during Ottoman rule. In keeping with Thai traditions, ‘Si Mai Thong’, which translates to ‘color of golden silk’ in Thai, is the suggested Thai name for the mocha coloration with the allelic designation *c* ^*m*^. ‘Wila Krung Thep’ is suggested for the name of the Si Mai Thong (mocha) colored cats. Wila is an older word for cat in the Thai language and Krung Thep means Bangkok, which is the discovery location of the novel coloration. The current allelic series for *TYR* variants in cats is: *C* (*full colour*) > *c* ^*b*^ (*Burmese*) = *c* ^*s*^ (*Siamese*) *> c*,* c* ^2^ (*albinos*). The mocha allele is likely co‐dominant to the Burmese allele and temperature sensitive, as displayed in the genotyped kittens. But as adults, the Burmese temperature‐sensitive allele may appear more influential in coloration. The mocha allele is also likely dominant to the Siamese (*c* ^*s*^) allele, as parentage‐inferred heterozygous kittens (*c* ^*m*^ *c* ^*s*^) displayed the mocha coloration instead of the otherwise expected Siamese coloration (Fig. 1c). However, not all allelic combinations have been observed, particularly combinations with the full albino alleles.

A variety of loss‐of‐function variants that cause complete albinism in *TYR* have been identified in various species. Temperature‐sensitive variants have been identified in mouse, cat, mink and human (Kwon *et al*. 1989; Giebel *et al*. 1991; Aigner *et al*. 2000; Benkel *et al*. 2009). These amino acid changes apparently reduce enzymatic activity in warmer regions of the animal's body, such as the torso. Full coloration is displayed at cooler regions, such as the ears, face, paws and tail, leading to the term ‘points’ of the Siamese cat. The sable coloration of the Burmese cat is less thermally labile, producing a darker coloration for the cat with full black coloration at the points. The mocha variant appears to interact with the temperature‐sensitive alleles but also produces a more even, lighter coloration and is less thermally labile when homozygous. This variant introduces two novel amino acids and deletes 39 amino acids of exon 2, which causes a 37‐amino‐acid deletion and does not include major functional domains (Fig. 2).

In conclusion, a tandem duplication in exon/intron 2 in *TYR*, which causes alternative splicing and results in c.820_936delinsAATCTC (p.Ile274_Leu312delinsAsnLeu), is associated with a novel coloration termed ‘mocha’ in cats. Furthermore, genotyping of 53 cats derived from Thailand identified two cats heterozygous for this variant. Therefore, this allele is polymorphic among cats in Thailand.

## Conflict of interest

The authors may receive supportive funds from a genetic testing laboratory that would offer this variant as a commercialized test in the future.

## Supporting information


**Figure S1** Pedigree depicting mocha coloration inheritance in Burmese cats.

**Figure S2** Gel electrophoresis of *TYR* exon 2 for mocha coloration.

**Figure S3** Partial nucleotide alignment of feline *TYR* and other species.

**Table S1** PCR primers for the amplification of feline *TYR*.

**Table S2** *TYR* exonic SNV genotypes in cats.

**Table S3** Genotyping of Thailand cat populations for the *TYR* mocha variant.

**Appendix S1** Materials and methods.

**Appendix S2** Sequence context of the mocha variant (*c* ^*m*^) and positions of the primers.
