# Supplementary material for: Mocha tyrosinase variant: a new flavour of cat coat coloration
Source: Anim Genet. 2019 Feb 4;50(2):182–6. doi: 10.1111/age.12765 (PMC6590430; doi:10.1111/age.12765)

Figure S1 Pedigree depicting mocha coloration inheritance in Burmese cats. Circles represent female and squares represent males. The cat with the arrow is the proband mocha-colored cat. Solid symbols indicate homozygous for c^m allele, half-filled indicate heterozygous for c^m allele, and open indicate wildtype for c^m allele (i.e., no c^m allele). Cats with ID numbers were genotyped for the *color* (*C*) locus alleles by Sanger sequencing, and they are listed below. The exon 2 duplication genotypes are presented as +/+ for homozygous duplication, +/- as heterozygous, and –/– as wildtype. The six cats with a dot in the upper right of symbol were Sanger sequenced for all five *TYR* exons.

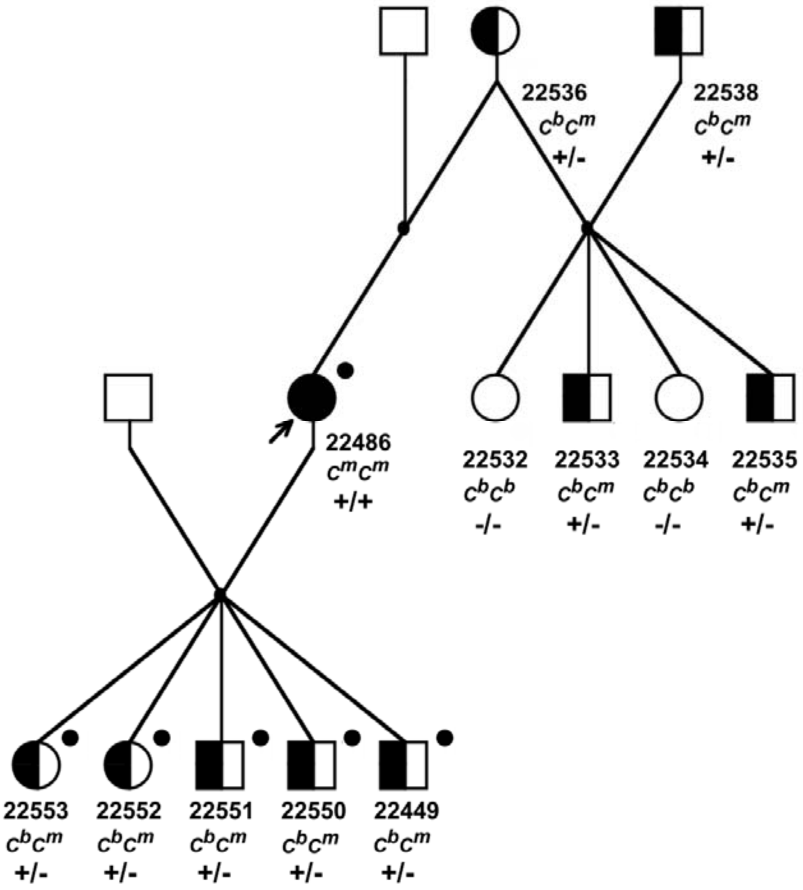

Supplement: Supplementary file 1 — Figure S1 Pedigree depicting mocha coloration inheritance in Burmese cats. [file AGE-50-182-s001.pdf]
