# Supplementary material for: Mocha tyrosinase variant: a new flavour of cat coat coloration
Source: Anim Genet. 2019 Feb 4;50(2):182–6. doi: 10.1111/age.12765 (PMC6590430; doi:10.1111/age.12765)

Figure S2 Gel electrophoresis of *TYR* exon 2 for mocha coloration. A 100-bp ladder is presented with the brightest band at 500 bp. (a) Homozygous variant (*) ~851 bp, a heterozygous carrier cat (**), a wildtype cat (†) ~688 bp, and a negative control (‡) for genomic DNA (b) cDNA amplicons for *TYR* exon 2 tandem duplication, amplified by primer 2 and primer 1–2 respectively. A 986 bp including exon 1–2 was expected and identified in the wildtype cat, whereas the cat with mocha coloration showed a smaller amplicon of 875 bp.

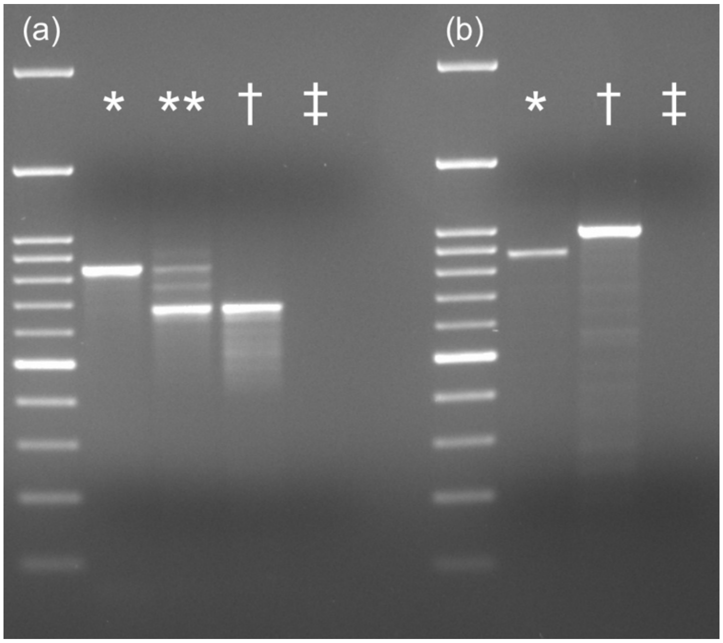

Supplement: Supplementary file 2 — Figure S2 Gel electrophoresis of TYR exon 2 for mocha coloration. [file AGE-50-182-s002.pdf]
