# Supplementary material for: Mocha tyrosinase variant: a new flavour of cat coat coloration
Source: Anim Genet. 2019 Feb 4;50(2):182–6. doi: 10.1111/age.12765 (PMC6590430; doi:10.1111/age.12765)

Figure S3 Partial nucleotide alignment of *TYR* in feline and other species. *TYR* nucleotide sequences are aligned from wildtype cat (*Felis catus*), mocha colored cat, dog [*Canis familiaris*; NC_006603.3 (CanFam3.1)], rabbit [*Oryctolagus cuniculus*; NC_013669.1 (OryCun2.0)], cow [*Bos Taurus*; NC_037356.1 (ARS-UCD1.2)], human [*Homo sapiens*; NC_000011.10 (GRCh38.p12)], mouse [*Mus musculus*; NC_000073.6 (GRCm38.p4)]. Intronic sequence is lowercase; exonic sequence is uppercase. Identical nucleotides to *Felis catus* are represented as dot (.). Nucleotides deleted in the mocha-coated cat are represented as a dash (–). Sequence is presented from the 3' portion of exon 1 to the 5' portion of exon 3. Green bars indicate additional intronic sequence. An asterisk (*) indicates the Siamese 'pointed' mutation (p.Gly302Arg). The mocha-coated cat has a 39 amino acid deletion (yellow highlight) and two amino acid insertion (pink highlight) caused by the presence of the new splicing acceptor site (tag) followed by the intronic 6 bp 5' of the duplicated partial exon 2 (blue highlight). Therefore, the mocha-coated cat has a 117-bp deletion concurrent with a 6-bp alternative transcription resulting in the c.820_936delinsAATCTC (p.Ile274_Leu312delinsAsnLeu) altered transcript.

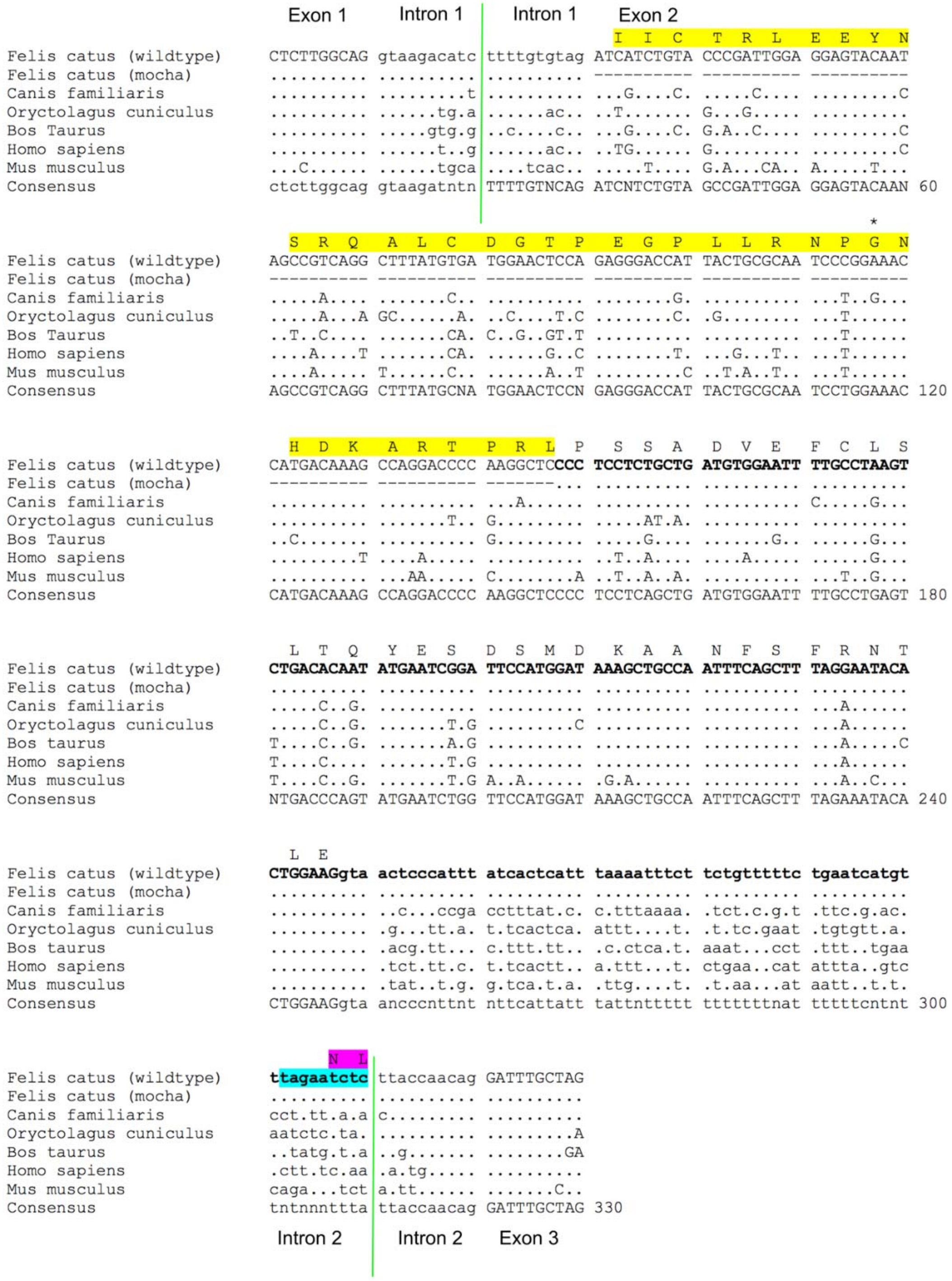

Supplement: Supplementary file 3 — Figure S3 Partial nucleotide alignment of feline TYR and other species. [file AGE-50-182-s003.pdf]
