# Supplementary material for: Mocha tyrosinase variant: a new flavour of cat coat coloration
Source: Anim Genet. 2019 Feb 4;50(2):182–6. doi: 10.1111/age.12765 (PMC6590430; doi:10.1111/age.12765)
Supplement: Supplementary file 4 — Table S1 PCR primers for the amplification of feline TYR. [file AGE-50-182-s004.pdf]

Table S1 PCR primers for the amplification of feline *TYR*.

TYR exon	Primer sequence (5'-3')	Size (bp)	Exon size (bp)	Annealing temp. (°C)
	**Genomic; forward/reverse**			
1	GGGACTGGTGTTATATAGGTCTTAGC<br>TAATAGAGGACCTTTGCTACTCTTGG	1076	1045	55
1-F2*	TCAATGTTTATGACCTCTTCGTCTGGA	448	1045	55
2	CTAGATTTTAACTTTCACTGCTTACGC<br>AAAATTCTGCCTTCTTAAACATATCTGG	688	217	55
2N†	CACCCAGATGCCTGTCAAC<br>TGGATTATGGTGTGCTGCTC	494	217	63
3	CCTACTACAGCATGAATTTTATTACTTTGAA<br>AATCCGATCTGAGACAACAAAGTACC	395	148	55
4	CATGTCTTGGAATTTTAAGATATGCTG<br>AATGATAAATTAAGGTTTGTAGTATGCTTTC	428	182	55
5	ACTAAGTAGCAGAGCTGACATTCAAAC<br>AATTCTGGTAACAGTCTTCACAAACC	504	761	55
	**cDNA**			
1-2	TCCGTGAAGACGAGGGTAAG<br>AGGCAAAATTCCACATCAGC	986		60
1-3/4	GCATCCTTCTTCTCCTCTTGG<br>TTCAAAAATACTGTCAACAAATGC	399		58
2/3-5	TGGAAGGATTTGCTAGTCCAC<br>GGGACTGCTCTTGGAACATC	656		58

*Primer 1 and primer 1-F2 use the same reverse primer for feline *TYR* amplification of exon 1. †Both primer 2 and primer 2N amplify the entire exon 2, but primer 2N was newly designed to sequence the entire duplicated region.
