# Supplementary material for: Mocha tyrosinase variant: a new flavour of cat coat coloration
Source: Anim Genet. 2019 Feb 4;50(2):182–6. doi: 10.1111/age.12765 (PMC6590430; doi:10.1111/age.12765)
Supplement: Supplementary file 5 — Table S2 TYR exonic SNV genotypes in cats. [file AGE-50-182-s005.pdf]

Table S2 *TYR* exonic SNV genotypes in cats.

		Mocha (c^m) c.820_936delinsAATCTC	Burmese (c^b) c.679G>T	Siamese (c^s) c.904G>A
Cat ID*	Phenotype‡	I274_L312delinsNL	p.G227W	p.G302R
22449	–	+/-	G/T	G/G
22450	–	+/-	G/T	G/G
22451	–	+/-	G/T	G/G
22452	–	+/-	G/T	G/G
22453	–	+/-	G/T	G/G
22486	Mocha	+/+	G/G	G/G
22532	Sable	-/-	T/T	G/G
22533	Burmocha	+/-	G/T	G/G
22534	Sable	-/-	T/T	G/G
22535	Burmocha	+/-	G/T	G/G
22536	Sable	+/-	G/T	G/G
22538	Sable	+/-	G/T	G/G
1	Light brown	+/-	G/T	G/G
2†	Tortoiseshell	-/-	G/T	G/G
3	Light brown	+/-	G/T	G/G
4	Light brown	+/-	G/T	G/G
5	Mocha	+/+	G/G	G/G
6	Light brown	+/-	G/T	G/G
7	Light brown	+/-	G/T	G/G
8	Mocha	+/+	G/G	G/G

*Cats 1 – 8 were genotyped by commercial services for Burmese and Siamese allele. †Parent of cat 3 and 4.

‡Phenotype was listed only when coat colors of the cats were supported by photographs.
