# Supplementary material for: Mocha tyrosinase variant: a new flavour of cat coat coloration
Source: Anim Genet. 2019 Feb 4;50(2):182–6. doi: 10.1111/age.12765 (PMC6590430; doi:10.1111/age.12765)
Supplement: Supplementary file 6 — Table S3 Genotyping of Thailand cat populations for the TYR mocha variant. [file AGE-50-182-s006.pdf]

Table S3 Genotyping of Thailand cat populations for the *TYR* mocha variant.

Population	No.	Coloration	Exon 2 dup
Thai	5	Chocolate-point	-/-
Khao Manee	20	White	-/-
Random bred	2	Unknown	+/-
Random bred	26	Unknown	-/-
