# Supplementary material for: Mocha tyrosinase variant: a new flavour of cat coat coloration
Source: Anim Genet. 2019 Feb 4;50(2):182–6. doi: 10.1111/age.12765 (PMC6590430; doi:10.1111/age.12765)
Supplement: Supplementary file 7 — Appendix S1 Materials and methods. [file AGE-50-182-s007.pdf]

Appendix S1

Supplemental materials and methods

Sampling and DNA isolation

Coat color of the cats were reported by the owners and/or supported by photographs. Breeding information, including mating types and coloration of offspring were provided by cat breeders. Breeding was conducted in North America from imported Thai cats. Buccal swab samples were provided by owners in accordance with an approved University of Missouri institutional Animal Care and Use Committee protocol (ACUC protocol # 8292). The pedigree relationships were confirmed by parentage analyses using short tandem repeat polymorphisms as previously described (Lipinski *et al.* 2007). Genetic testing by a commercial laboratory (Veterinary Genetics Laboratory, University of California – Davis, Davis, CA, USA) suggested the cats were carriers for the *TYR* variant for sable coloration of the Burmese cat (c^b = c.679G>T) and wildtype for the other known *TYR* variants (c^s = c.904G>A [*siamese*], *c* = c.938delC [*albino*], c^2 = c.1204C>T [*albino*]) (Lyons *et al.* 2005; Schmidt-Küntzel *et al.* 2005; Imes *et al.* 2006; Abitbol *et al.* 2017). Genomic DNA was extracted from buccal swabs using QIAamp DNA mini kit (Qiagen, Valencia, CA, USA). For genotyping in Thailand cats, archival DNAs were available from 53 cats originally from Thailand including 20 Khao Manee cats, five cats of the developing "Thai" breed, and 28 random bred. The random bred cats were sampled from the streets of Bangkok, Thailand in 2009.

Mutation detection and sequencing

Primers for *TYR* were designed to flank the intron/exon boundaries for each of the five exons based on the publicly available sequence of cat assembly v9.0 (Felis_catus_9.0) using Primer3Plus

(http://www.bioinformatics.nl/cgi-bin/primer3plus/primer3plus.cgi/). Primer information was summarized in Table S1 and locations were indicated in Appendix 2. PCR was performed with Choice-Taq DNA Polymerase (Denville Scientific Inc, Holliston, MA, USA) on a Veriti 96-Well Thermal Cycler (Applied Biosystems, Foster City, CA, USA) in a total volume of 25 µl, including 2.5 µl of 10x PCR Buffer (the final concentration of 1.5 mM $MgCl_2$) for ChoiceTaq DNA Polymerase (Denville), 0.2 µl of 25mM each dNTP mix, 1.25 µl of each primer (10 pmol/µl), and 0.25 µl (1.25 U) of Choice-Taq DNA Polymerase (Denville) and 1–5 µl of DNA concentration of ~10–50 ng. PCR cycling conditions included the following steps: initial denaturation (94 °C for 3 min), followed by 35 cycles of denaturation at 94 °C for 45 s, annealing for 30 s, and extension at 72 °C for 1 min, and then a final extension at 72 °C for 10 min. PCR products were separated by electrophoresis using 1.5% agarose gels at 80 V for 60–90 min. Amplicons were excised from the gel and purified using QIAquick Gel Extraction Kit (Qiagen). Purified products were sequenced bi-directionally at the University of Missouri DNA Core using an Applied Biosystems 3730xl DNA Analyzer (Applied Biosystems) with BigDye Terminator v3.1 Cycle Sequencing Kit (Applied Biosystems). Exon 1 was sequenced using only the forward primer due to the presence of an adenosine repeat sequence near the reverse primer. Sequencing results were analyzed using 4 peaks v1.8 for Mac OS X (Nucleobytes, Aalsmeer, the Netherlands; https://nucleobytes.com/) and aligned using the CLC Sequence Viewer v7.7.1 (CLC Bio, Aarhus, Denmark; https://www.qiagenbioinformatics.com/products/clc-sequence-viewer/).

RNA extraction and cDNA sequencing

One cat with mocha coloration was presented to a local veterinary clinic (Ashland Road Animal Clinic, Mansfield, OH, USA) for physical examination and skin biopsy collection. All procedures

were conducted with informed consent and conducted by a veterinarian and trained veterinary technicians. Local anesthesia was achieved using 2% lidocaine hydrochloride (Bimeda-MTC Animal Health, Cambridge, Canada) at the biopsy site. A 6 mm biopsy punch (Miltex, York, PA, USA) sample was collected from the caudal dorsocervical area from the mocha cat. The skin sample was cut into three portions, and one portion was snap frozen in liquid nitrogen and two portions were immersed in RNAlater (Qiagen), immediately. As a control, an archived frozen skin sample was available from one non-mocha pointed cat (c^sc^s).

Reverse transcription PCR (RT-PCR) was performed on isolated total RNA to determine the effect of the identified variant on the *TYR* transcript. Total RNA was extracted from the skin samples using PureLink RNA Mini Kit (Invitrogen, Carlsband, CA, USA) according to manufacturer's protocol. Reverse transcription was performed using SuperScript III (Invitrogen) with Oligo(dT)20 Primer (Invitrogen) according to manufacturer's protocol. PCR was performed using the synthesized cDNA and the primer sets designed using Primer3Plus (Table S1), and PCR was performed using the same procedure described above except for 1 min for the initial denaturation time. The size of the RT-PCR product was confirmed on an agarose gel, and fragment isolation and sequencing was performed as described above.

Prediction of structures

Human *TYR* contains a signal peptide (1–18 AA), an epidermal growth factor (EGF)-like domain (61–112 AA), copper-binding sites A (180–211 AA) and B (363–390 AA), and transmembrane domain (476–496 aa) (Oetting, 2000). Those structures' positions were checked using PROSITE (https://prosite.expasy.org/), SignalP 4.1 Server (http://www.cbs.dtu.dk/services/SignalP/), TMHMM Server v. 2.0 (http://www.cbs.dtu.dk/services/TMHMM/). In addition to known feline

TYR variants, several temperature-sensitive *TYR* variants in other mammals are depicted in Fig. 2 (Kwon *et al.* 1989, Giebel *et al.* 1991, Aigner *et al.* 2000, Benkel *et al.* 2009).

Genotyping in Thailand cats

Three cat populations including cats representing the Khao Manee and Thai breeds, and random bred cats from Thailand were genotyped for the identified *TYR* variant. PCR was performed using the primers targeting the exon 2 duplicated region (Table S1) using the same conditions as the mutation detection described above, and amplicon size was confirmed by agarose gel electrophoresis. When the heterozygous amplicon was detected, Sanger sequencing was performed to confirm the sequence on both PCR products.

Assay development

Each PCR reaction contained 3 µl of template DNA isolated under previously described conditions (Oberbauer *et al.* 2003). Product amplification occurred with the following thermal profile: 95 °C 3 min, 85 °C 5 min, 5 cycles of 95 °C 1 min, 58 °C 30 sec, 72 °C 30 sec, 28 cycles of 95 °C 45 sec, 58 °C 30 sec, 72 °C 30 sec, followed by 1 cycle of 72 °C for 30 min and a 10 °C infinite hold. Each 25 µl reaction contained 1 µM of each primer (Forward = Fam-AACCATGACAAAGCCAGGAC, flanking reverse = TCTTAAACATATCTGGGACCAG and splice site specific reverse = AGGAGGGGAGATTCTAAACAT), 1X reaction buffer (Denville), 2.5 mM $MgCl_2$, 1.0 mM dNTP, and 1.0 U of Choice-Taq DNA Polymerase (Denville). Template DNA and primers were initially denatured for 3 minutes at 95 °C in a 10 µl volume with the remaining 15 µl of reaction mix being added during the 85 °C pre-cycling soak. PCR amplicons were visualized on an Applied Biosystems 3730xl DNA Analyzer (Applied Biosystems) and

analyzed using STRand software (Toonen & Hughes 2001). The wild-type allele was 226 bp while the affected allele was 200 bp. If only the forward and flanking reverse primers are used, a 389 bp product is observed in animals possessing either one or two copies of the duplication. However, a 10-15x relative decrease of the larger product is observed in heterozygous individuals. The location of the primers for this assay were indicated in Appendix 2.

References

Abitbol M., Bosse P., Grimard B., Martignat L. & Tiret L. (2017) Allelic heterogeneity of albinism in the domestic cat. *Anim Genet* **48**, 127-8.

Aigner B., Besenfelder U., Muller M. & Brem G. (2000) Tyrosinase gene variants in different rabbit strains. *Mamm Genome* **11**, 700-2.

Benkel B.F., Rouvinen-Watt K., Farid H. & Anistoroaei R. (2009) Molecular characterization of the Himalayan mink. *Mamm Genome* **20**, 256-9.

Giebel L.B., Tripathi R.K., King R.A. & Spritz R.A. (1991) A tyrosinase gene missense mutation in temperature-sensitive type I oculocutaneous albinism. A human homologue to the Siamese cat and the Himalayan mouse. *J Clin Invest* **87**, 1119-22.

Imes D.L., Geary L.A., Grahn R.A. & Lyons L.A. (2006) Albinism in the domestic cat (Felis catus) is associated with a tyrosinase (TYR) mutation. *Anim Genet* **37**, 175-8.

Kwon B.S., Halaban R. & Chintamaneni C. (1989) Molecular basis of mouse Himalayan mutation. *Biochem Biophys Res Commun* **161**, 252-60.

Lipinski M.J., Amigues Y., Blasi M., Broad T.E., Cherbonnel C., Cho G.J., Corley S., Daftari P., Delattre D.R., Dileanis S., Flynn J.M., Grattapaglia D., Guthrie A., Harper C., Karttunen P.L., Kimura H., Lewis G.M., Longeri M., Meriaux J.C., Morita M., Morrin-O'donnell R

C., Niini T., Pedersen N.C., Perrotta G., Polli M., Rittler S., Schubbert R., Strillacci M.G., Van Haeringen H., Van Haeringen W. & Lyons L.A. (2007) An international parentage and identification panel for the domestic cat (Felis catus). *Anim Genet* **38**, 371-7.

Lyons L.A., Imes D.L., Rah H.C. & Grahn R.A. (2005) Tyrosinase mutations associated with Siamese and Burmese patterns in the domestic cat (Felis catus). *Anim Genet* **36**, 119-26.

Oetting W.S. (2000) The tyrosinase gene and oculocutaneous albinism type 1 (OCA1): A model for understanding the molecular biology of melanin formation. *Pigment Cell Res* **13**, 320-5.

Schmidt-Küntzel A., Eizirik E., O'Brien S.J. & Menotti-Raymond M. (2005) Tyrosinase and tyrosinase related protein 1 alleles specify domestic cat coat color phenotypes of the albino and brown loci. *J Hered* **96**, 289-301.

Oberbauer A.M., Grossman D.I., Eggleston M.L., Irion D.N., Schaffer A.L., Pedersen N.C. & Belanger J.M. (2003) Alternatives to blood as a source of DNA for large-scale scanning studies of canine genome linkages. *Vet Res Commun* **27**, 27-38.

Toonen R.J. & Hughes S. (2001) Increased throughput for fragment analysis on an ABI PRISM 377 automated sequencer using a membrane comb and STRand software. *Biotechniques* **31**, 1320-4.
