# Supplementary material for: Mocha tyrosinase variant: a new flavour of cat coat coloration
Source: Anim Genet. 2019 Feb 4;50(2):182–6. doi: 10.1111/age.12765 (PMC6590430; doi:10.1111/age.12765)
Supplement: Supplementary file 8 — Appendix S2 Sequence context of the mocha variant (c m) and positions of the primers. [file AGE-50-182-s008.pdf]

Appendix S2

Sequence context of the mocha variant (c^m) and positions of the primers

cacccagatgcctgtcaacagtttacttaaaatatgaacctcttgatgataatctctagccac

aactcctttaacataaaggtttttgtgtagATCATCTGTACCCGATTGGAGGAGTACAATAGCC

GTCAGGCTTTATGTGATGGAACTCCCCGAGGGGACCATTACTGCGCAATCCCGG|AACCATGACA

AAGCCAGGACCCCAAGGCTC**CCCTCCTCTGCTGATGTGGAATTTTGCCTAAGTCTGACACAATA**

TGAATCGGATTCCATGGATAAAGCTGCCAATTTCAGCTTTAGGAATACACTGGAAGgtaactcc

catttatcactcatttaaaatttcttctgtttttctgaatcatgtttagaatctcCCCTCCTCT

GCTGATGTGGAATTTTGCCTAAGTCTGACACAATATGAATCGGATTCCATGGATAAAGCTGCCA

ATTTCAGCTTTAGGAATACACTGGAAGgtaactcccatttatcactcatttaaaatttcttctg

tttttctgaatcatgtttagaatctcttatccaaaaccctggtcccagatatgtttaagaaggc

agaattttctgaataaggaaattggcctattgcctatgtggccaataatcctcacagagtctga

gcagcacaccataatcca

The original sequence was retrieved from Felis_catus_9.0 (NC_018732.3: 45898489–45898983). The mocha tandem duplication region was inserted. Exonic region (Exon 2) is capitalized. Intronic region is indicated as lower cases. Duplicated sequence found in mocha cats is in bold, and is repeated as underlined sequences. Primer sequences used for PCR and Sanger sequencing of feline *TYR* are highlighted in yellow. Primers used for the assay developed for the c^m variant detection are highlighted as follows: forward primer with purple, flanking reverse primer with green, and splice-site specific primer with blue. Please note, the cat assembly v9.0 (Felis_catus_9.0) has two insertions and one deletion in the region (NC_018732.3: 45898489–45898983) as indicated by red highlights and a black vertical line, respectively. In addition, *TYR* reference sequence XM_003992642.4, which is from Felis catus Annotation Release 104, has one insertion and one deletion as indicated by the second red highlight and a black vertical line, respectively. Both of them are incorrect annotations. Accurate sequences for the *TYR* exon 2 region include these accessions: AH014863.2, AY743346.1, AY743347.1, and AY743348.1.
